# Adaptation and validation for Chilean Spanish of the checklist individual strength (CIS) in Chilean workers

**DOI:** 10.1186/s12889-026-28872-2

**Published:** 2026-08-01

**Authors:** Emilio Moyano-Díaz, Héctor Vargas-Garrido, Doris Méndez-Campos

**Affiliations:** Faculty of Psychology, University of Talca, Talca, Chile

**Keywords:** Checklist Individual Strength, Psychometrics, Work fatigue, Health, Absenteeism, Job satisfaction

## Abstract

**Background:**

Work fatigue (WF) is a complex physical and mental exhaustion caused by multiple factors. It is a growing occupational and health phenomenon in contemporary society. Thus, there is a need for validated and adapted instruments in different languages for an adequate assessment of this pressing public health problem.

**Methods:**

In this study the psychometric properties of the original Anglo-American version of the 20-item CIS were examined in 686 Chilean workers in the security and education employment sectors (Spanish Latin American version).

**Results:**

A 16-item version (CIS-16R) was obtained, with adequate reliability (Cronbach’s alpha of 0.93 and 0.86) and content validity (*r* = .76 with the 3D-WFI). Four of its original items were eliminated, as they needed to exceed the item-total correlation cut-off point adequately. An exploratory factor analysis yielded a bi-factor structure (and not four as in its original version), which we named general and physical fatigue, respectively.

**Conclusions:**

The Spanish-adapted version of the CIS is proposed. In addition, the weight of job demands and other ergonomic and environmental variables in generating WF were identified. Among the effects of WF, self-assessments of reduced health, higher absenteeism, lower job satisfaction, and lower sleep quality were observed.

## Background

Workers are exposed to several occupational hazards, including work fatigue (WF). WF is challenging to conceptualize due to its breadth, the variety of its causal factors, and its behavioral manifestations. The first effect of WF is a decrease in performance. Reports indicate that fatigue symptoms affect 10 to 33% of the world’s population, with significant costs to the healthcare system. WF generally manifests as exhaustion, excessive sleepiness, and general tiredness and can be considered a warning or signal to the body that it should rest [1, 2].

WF has been associated with inadequate information processing, mistakes in the execution of tasks, decreased performance, and increased risk-taking, all of which can lead to accidents and occupational diseases. It can manifest as usual or acute fatigue (no more than 1 month) or chronic or pathological (6 or more months). Recovery from the former is achieved with rest. At the same time, the latter requires more than just regular rest, as it manifests extreme fatigue and the impossibility for affected workers to perform their usual activities and is usually associated with co-morbidities or concomitant diseases [3].

Among the factors identified as causing WF are sustained stress at work due to repetitive task demands, continuous muscular work, mental overload or underload, vibrations, lighting, noise, temperature, or poor physical-environmental conditions in the workplace [1], sleep deprivation (night work shifts), poor psychosocial conditions of autonomy, skills needed to perform the job well, and interpersonal relationships [4, 5].

Sustained stress can lead to burnout, and some authors consider WF a component of the latter. Both cause sickness absence from work and share common causes and manifestations regarding prolonged stress and the job demands mentioned above [6]. The COVID-19 pandemic was a new source of stress and burnout and, thus, of WF; it increased anxiety in workers due to the fear of being infected or due to an unusual exposure of many hours to screens by teleworking. It has been internationally and locally reported that healthcare workers in health services or ‘front-line’ care for COVID-19 experienced fatigue and emotional exhaustion. The same was true for the general population, who, as an effect of quarantines, experienced negative symptoms, including post-traumatic stress symptoms, confusion, and anger [7].

Sagherian et al. [8] showed that nursing staff reported sub-threshold insomnia, moderate to high chronic fatigue, high acute fatigue, and low to moderate inter-shift recovery. Staff involved in the care of COVID-19 patients worked more than 40 h per week, skipped breaks, and experienced increased emotional exhaustion and depersonalization, moderate psychological distress, and high post-traumatic stress. Nurses who cared for COVID-19 patients scored significantly worse on almost all measures than their coworkers on sleep deprivation, fatigue, and multiple psychological problems.

WF might even better explain occupational accidents associated with shift work and sleep problems as a general consequence. Thus, WF would be the closest antecedent to occupational accidents or diseases. The accident registrar in Chile does not include information related to WF as an associated or causal factor of accidents or occupational diseases, nor of possible incidents associated with fatigue in work environments [9]. Consequently, strategies to identify and manage this type of occupational risk are unavailable.

WF is a phenomenon that has been present in work contexts since ancient times and is seen as a functional state of protective significance, which produces an unpleasant physical sensation with cognitive and emotional components [3]. A meta-analysis showed that 90% of shift workers experienced fatigue and sleepiness in their workplace [10]. Although today’s work demands increasingly expanded mental and emotional capacities, there is still work of a physical or muscular nature, where repetitive movements are related to fatigue due to the dynamic contractions executed by the worker, and where work environments below 19 °C and above 28 °C have a significant effect on muscle fatigue [11].

A systematic review of the relationship between sickness absence and WF, including 16 studies, showed that 14 provided evidence of the relationship between both variables [8]. WF costs employers in the United States an estimated $136 billion yearly [12] in insurance and other costs due to absenteeism, illness, or workers’ accidents.

González et al. [13] showed that workers exposed to long working hours of more than 10 h suffer a direct impact on mental functions concerning the execution of their natural abilities, indicating that the higher the professional level, the more complex the responsibilities tend to be, with a consequent greater tendency to experience mental WF. A recent systematic review of physical fatigue shows that the various intervention efforts to mitigate it have not been very effective [14].

In Chile, WF has been measured in high-altitude mining workers and healthcare personnel. To the best of our knowledge, the first article published on the subject, using the Checklist Individual Strength (CIS) by Beurskens et al. [15] corresponds to Vera et al. [16] in high-altitude miners (*n* = 120). They found a prevalence of physical and cognitive fatigue of 41.7% and significant associations between acute mountain sickness, physical fatigue, cognitive fatigue, and different psychosocial and occupational factors. The researchers concluded that acute mountain sickness was the predictor of WF with the most significant weight or importance, compared to the rest of the psychosocial and occupational variables studied, and that the greater the physical fatigue, the lower the job satisfaction.

In a second study, Seguel and Valenzuela [3], using the CIS version of Vera et al. [16], in nursing and paramedics staff (*n* = 343) at two Chilean hospitals reported a Cronbach’s alpha of 0.78 and a correlation of WF with burnout syndrome (which is also a cause of absenteeism), specifically in the emotional exhaustion dimension (*Rho* = 0.680, *p* = .000).

The most recent Chilean study of WF using the CIS [17] was also conducted in the health sector (110 nursing assistants). The association between WF and absenteeism in a highly complex hospital was analyzed. It was observed that the predominant absenteeism interval was 11 to 29 days and a significant association between physical fatigue (OR = 1.05; *p* = .04) and seniority (OR = 1.08; *p* = .001) with absenteeism was found; thus, physical fatigue and seniority in that clinical service represented a significant risk factor for absenteeism.

The three previous studies that measured WF using the CIS administered only 15 of its original 20 items. Vera et al. [16] did not explain or detail why they proposed a 15-item version, nor did they explain which items were eliminated and why. They derived two subscales from their study: “one assesses Subjective Perception of Physical Fatigue (Cronbach’s α = 0.85), and the second assesses Subjective Perception of Cognitive Fatigue (Cronbach’s α = 0.78)” (p. 91). Correlational analyses were performed on both subscales with the stress and depression scales showing positive and significant results, thus supporting the concurrent validity of that version. In Seguel and Valenzuela [3], without seeking psychometric objectives, the exact Vera et al. [16] 15-item CIS version was used in a study with 343 health workers (101 nurses, 235 paramedics, and seven did not report their position). A Cronbach’s alpha of 0.78 was reported. In turn, the study by Luengo et al. [17], also without psychometric objectives, was conducted with a small sample and the authors did not report the reliability of the CIS.

Considering the limitations associated with the use of the 15-item version of the CIS in previous studies and the lack of validated instruments in Spanish to measure WF in Chilean workers, this research aims to address this gap by conducting a psychometric study aimed at adapting and advancing the validation of the original 20-item instrument, the CIS20. Conversely, the aim was to identify factors producing WF and their effects on workers. Additionally, we sought to both verify the hypothesis of a positive relationship between WF and absenteeism and a negative association between job satisfaction and health self-assessment in the direction of previous literature and explore other relationships with personal and environmental variables in the workplace.

## Method

### Sample

The study employed a non-probabilistic sampling method. The sample consisted of 686 Chilean workers who voluntarily completed a survey on WF and belonged to three organizations—one from the security sector (501 participants) and two from the education sector (185 participants). Regarding geographical distribution, the subsample from the security sector exhibited broad national coverage, including participants from 12 of the country´s 16 regions. In contrast, the subsample from the education sector was drawn from a single region. This sample of workers was used to comply with the recommended criteria for a psychometric study regarding the ratio of the number of items in the instrument versus number of participants (1:10 or 1:15).

### Instruments

Four instruments were used in a voluntary simultaneous application with prior informed consent from individuals (Scientific Ethics Committee of the University of Talca, File 43-2021). In addition, socio-demographic and family information, work conditions (shifts, number of employers, remuneration), work accident record, and job satisfaction information were requested. The four instruments:

i) Checklist Individual Strength (CIS) [15]. It measures work fatigue by 20 items with a 7-option Likert response format (1 = yes, this is true, up to 7 = no, this is not true) distributed unequally in four subscales that constitute its factorial architecture: the subjective feeling of fatigue (eight items), concentration (five items), motivation (four items), and physical activity (three items). Although in its original English version there is no report of its reliability that emphasizes its discriminative power between groups of different fatigue levels, its German version had a Cronbach’s alpha reliability of 0.90 for the general scale, and the subscales ranged between 0.92 and 0.83 [18]. As indicated in Table 2, the inversion of some items (r) leads to the fact that the higher the score, the higher the level of fatigue.
ii) Health-Related Quality of Life Index (HRQLI), by Hennessy et al. [19], Spanish version. It measures general self-assessment of health by a single item with a 5-choice response format (poor, fair, good, very good, or excellent), and asks for the number of days in which the respondents felt ill in their physical health, the same for their mental health, and, finally, for how many days in the last month they were absent from work due to illness. Its reliability in the Chilean population is 0.66 [20].
iii) The Three-Dimensional Work Fatigue Inventory (3D-WFI) by Frone and Tidwell [4]. It consists of 18 questions divided into three parts of six questions each, evaluating physical, mental, and emotional work fatigue, respectively, during the last 12 months. In all three dimensions, work fatigue increases with higher scores. Its response format is Likert-type with five alternatives (every day, at least once a week, at least once a month, less than once a month, or never). Its Cronbach's alpha reliability in the original version for fatigue due to physical work is 0.94, mental work 0.95, and emotional work 0.96. The Spanish version was used [21].
iv) A 10-item scale to measure the perception of personal demands of the workplace (five items) and the workplace's physical-environmental characteristics (five items).

### Procedure

Following the guidelines of the International Test Commission [22, 23], two bilingual English-Spanish academics independently translated the CIS into Spanish. Later, they agreed on a version they considered more in line with the original. They used colloquial language in Chilean Spanish, thus facilitating the understanding of the respondents’ educational or cultural differences. Previous Chilean versions were also considered in translating the CIS [3, 16, 17].

### Analysis plan

First, the reliability of the CIS was determined using conventional criteria of item-total correlation to assess the relevance of the inclusion of each of its items to provide evidence that will allow subsequent researchers to carry out confirmatory factor analysis procedures. Items with item-scale correlation values of less than 0.30 will be eliminated from the final validated instrument. An exploratory factor analysis of the CIS was then carried out. Thus, Cronbach’s alpha and McDonald’s omega were used to calculate the reliability of the CIS scale. To verify the factorial structure of the instrument, an exploratory factor analysis procedure was carried out using the maximum likelihood extraction method and varimax rotation with Kaiser-varimax rotation. A bivariate correlation analysis of the CIS scale and its different sub-dimensions with the 3D-WFI instrument was carried out to analyze concurrent validity. Finally, linear regression analyses with the stepwise method were performed to identify the weight of possible variables that influence WF and others that are affected by it.

## Results

The average age of the participants was 41 years (SD = 11.98), while 64.6% were male and 35.4% female. In the present administration of the CIS to 686 Chilean workers in the security and education sectors, a Cronbach’s alpha of 0.93 was obtained. In the sample, 37.6% of the participants reported a medium (3.5) to moderate (up to 7) level of WF.

### CIS means and standard deviations

Some descriptive statistics of our CIS application are presented in Table 1. The average WF in the large sample was of a medium level, almost 3 out of 7, indicating a medium to low grade of WF in the sample, with a considerable degree of variability.

**Table 1:** Descriptive statistics for the CIS application (*n* = 686)

Mean	Median	Mode	SD	Minimum	Maximum	Skewness	Kurtosis
2.93	2.80	1.00	1.32	1	6.8	0.29	− 0.86

### Reliability and validity of the CIS

The mean, standard deviation, and corrected item-total correlation values are shown in Table 2. The highest mean value corresponded to Item 1, “I feel tired” (4.37), and the lowest to the item 5, “I feel like doing all kinds of nice things” (1.60). As we obtained item-scale correlation values of less than 0.30 for four items out of 20, we eliminated them from the final instrument (values italicized in Table 2).

**Table 2:** Averages, deviations, and corrected item-total correlation values for the CIS-20R (Spanish version)

Item	M	SD	CIC*
1. Me siento cansado. (r)	4.37	2.42	0.74
2. Me siento muy activo.	3.16	2.01	0.69
3. Pensar me cuesta trabajo. (r)	2.61	2.01	0.68
4. Físicamente me siento agotado. (r)	4.04	2.37	0.74
5. Tengo ganas de hacer cosas agradables.	1.60	1.25	*0.26*
6. Me siento en forma.	3.36	2.11	0.68
7. Hago muchas cosas durante el día.	2.09	1.64	*0.13*
8. Cuando estoy haciendo algo puedo concentrarme sin problemas.	2.44	1.84	0.66
9. Me siento débil. (r)	3.01	2.19	0.76
10. No hago mucho durante el día. (r)	2.28	1.91	*0.24*
11. Puedo concentrarme bien.	2.44	1.81	0.71
12. Me siento descansado.	4.11	2.27	0.64
13. Tengo problemas para concentrarme. (r)	3.00	2.17	0.71
14. Siento que estoy en mala condición física. (r)	3.82	2.35	0.64
15. Tengo muchos planes.	2.16	1.65	*0.22*
16. Me canso muy rápido. (r)	3.15	2.13	0.76
17. Tengo un bajo rendimiento. (r)	2.15	1.69	0.57
18. No tengo ganas de hacer ninguna cosa. (r)	2.41	2.00	0.68
19. Mis pensamientos se enredan fácilmente. (r)	2.59	2.05	0.66
20. Físicamente siento que estoy en buena forma.	3.60	2.26	0.64

Italicized values represent item-total correlation values of less than 0.30

* CIC:corrected item-total correlation value

Reliability indices were obtained for the total scale (*a* = 0.92, Ω = 0.92), for the subjective fatigue subscale (*a* = 0.91, Ω = 0.91), the concentration subscale (*a* = 0.89, Ω = 0.89), the motivation subscale (*a* = 0.61, Ω = 0.61), and the physical activity subscale (*a* = 0.49, Ω = 0.49).

Exploratory factor analysis was performed on the 16 items that exceeded the item-scale correlation cut-off, using maximum likelihood extraction and Kaiser-varimax rotation. Both the KMO indicator (0.94) and Bartlett’s test of sphericity (*x*^2^ = 7644.29, *p* = .001) suggested that the data were suitable for factor analysis.

The results show that the 16 items of the CIS-20R instrument are grouped into two factors, which we called “general fatigue” (*a* = 0.93, Ω = 0.94), and “physical fatigue” (*a* = 0.86, Ω = 0.87), respectively, explaining 58% of the variance. The Rotated Factors Matrix obtained for the instrument is shown in Table 3.

**Table 3:** CIS exploratory factor analysis results (Rotated Factors Matrix)

Item	Factor 1*	Factor 2**
1. Me siento cansado. (r)	0.62	
2. Me siento muy activo.	0.51	
3. Pensar me cuesta trabajo. (r)	0.76	
4. Físicamente me siento agotado. me siento agotado. (r)	0.62	
5. Tengo ganas de hacer cosas agradables.		
6. Me siento en forma.		0.83
7. Hago muchas cosas durante el día.		
8. Cuando estoy haciendo algo puedo. concentrarme sin problemas.	0.68	
9. Me siento débil. (r)	0.66	
10. No hago mucho durante el día. (r)		
11. Puedo concentrarme bien.	0.72	
12. Me siento descansado.		0.52
13. Tengo problemas para concentrarme. (r)	0.79	
14. Siento que estoy en mala condición física. (r)		0.77
15. Tengo muchos planes.		
16. Me canso muy rápido. (r)	0.61	
17. Tengo un bajo rendimiento. (r)	0.54	
18. No tengo ganas de hacer ninguna cosa. (r)	0.63	
19. Mis pensamientos se enredan fácilmente. (r)	0.70	
20. Físicamente siento que estoy en buena forma.		0.79

* Factor 1, general fatigue: 34% explained variance; ** Factor 2, physical fatigue: 24% explained variance

In addition, to support the construct validity of the CIS, the correlation of the CIS with the 3D-WFI instrument for measuring work fatigue adapted and validated for use in Chile [21] showed a significant bilateral positive correlation of 0.76 (*p* < .001).

### Predictive relationship between different work variables and CIS work fatigue

An analysis to identify the weight of some variables that might predict WF is presented in Table 4.

**Table 4:** Regression of predictor variables on CIS work fatigue

Predictor variables	Unstandardized and standardized betas on WF CIS subscales							
	General				Physical			
In your job, you are exposed to…	B	*SE* B	*β*	*t*	B	*SE* B	*β*	*t*
Heat and noise most of the time	-	-	− 0.01	-	-	-	− 0.01	-
Repetitive or monotonous tasks	0.10	0.04	0.09	2.30*	-	-	− 0.04	-
Making efforts to control emotions	0.35	0.04	0.32	8.08**	− 0.26	0.05	− 0.20	4.95**
Permanent concentration	0.14	0.04	0.13	3.40**	− 0.17	0.05	− 0.13	3.34**
Taking care of others	-	-	− 0.03	-	-	-	− 0.02	-
Harsh environmental conditions (outdoors, on the street, etc.)	-	-	− 0.04	-	-	-	− 0.07	-
Wearing uniforms, suits, or specific clothing	− 0.20	0.03	− 0.22	-6.00**	− 0.25	0.04	− 0.23	-6.19**

General subscale adjusted *R*^*2*^ = 0.21: stepwise method; *R*^*2*^ = 0.21, *F*(4, 589) = 40.87, *p* < .001; physical subscale adjusted *R*^*2*^ = 0.12: stepwise method; *R*^*2*^ = 0.13, *F*(3, 590) = 29.99, *p* < .001

* *p* < .05; ** *p* < .01

It was observed that jobs that require emotional control (i.e., “making efforts to control emotions”) or permanent concentration (i.e., “requires permanent concentration”) positively affected WF in its two sub-dimensions. At the same time, “wearing uniforms, suits, or special clothing” decreases physical fatigue and general fatigue in this sample. Finally, performing “repetitive or monotonous tasks” affected the general fatigue dimension, not the physical one.

A further regression analysis, using the stepwise method, related personal socio-demographic factors that might predict fatigue (gender, age, workload, and taking work home). The result showed significant relationships, except for age. The percentage of variance explained here reached 23% (Table 5).

**Table 5:** Regression of sociodemographic and workload variables on work fatigue (Overall CIS-20R)

Constant	B	SE B	β	t
	1.54	0.17		9.03**
Workload	0.47	0.05	0.33	8.63**
Gender (male)	− 0.51	0.10	− 0.18	-5.14**
Taking work home	0.20	0.04	0.16	4.26**

Adjusted *R*^*2*^ = 0.23: stepwise method; *R*^*2*^ = 0.23, *F*(3, 596) = 61.39, *p* < .001

* p < .05; ** *p* < .01

A regression analysis of different work variables in the proposed WF CIS-16R measure—sleep quality, occupational accidents, commuting accidents, job satisfaction, self-perceived general, physical, and mental health, and absenteeism (due to health problems)—showed that job satisfaction negatively predicted participants’ general fatigue. Participants with higher job satisfaction reported lower levels of general work fatigue. Likewise, negative self-perceived physical health (i.e., “Thinking now about your physical health, which includes physical illness and injury, during how many days during the past 30 days was your physical health not good? Indicate the number of days”) significantly predicted participants’ physical fatigue (see Table 6).

**Table 6:** Regression of work variables on work fatigue (general fatigue and physical fatigue per the CIS-16R version)

CIS-16R Subdimension		B	SE B	β	t
General fatigue	Constant	9.27	1.45		6.35*
	Job satisfaction	-1.62	0.35	− 0.95	-4.52*
Physical fatigue	Constant	2.24	0.18		12.08**
	Self-perceived physical health	0.16	0.03	0.95	4.64*

The variables excluded for general fatigue were sleep quality, occupational accidents, commuting accidents, self-perceived general, physical, and mental health, and absenteeism. The variables excluded for physical fatigue were job enjoyment, sleep quality, occupational accidents, commuting accidents, self-perceived general and mental health, and disability. General fatigue adjusted *R*^*2*^ = 0.86: stepwise method; *R*^*2*^ = 0.91, *F*(1, 2) = 20.48, *p* = .046; physical fatigue adjusted *R*^*2*^ = 0.87: stepwise method; *R*^*2*^ = 0.91, *F*(1, 2) = 21.59, *p* = .043

* p < .05; ** *p* < .01

## Discussion

From its original version of 20 items, in this study the CIS was reduced to 16 items to optimize its psychometric performance during a Spanish application with a large sample of Chilean workers. This version can now be accurately referred to as the CIS-16R. The Cronbach’s alpha reliability coefficient was high, indicating adequate reliability. The analysis of its factorial structure did not reproduce the four-factor structure initially proposed in its Anglo-American version. Previous works in Spanish have also reported problems with entirely using the CIS-20 in Spanish, opting for versions with fewer items (15) and factors (2) [16]. In the present study, it was observed that four items of the CIS-20R achieved a test-item correlation of less than 0.30, which suggested their elimination. Subsequently, an exploratory factor analysis revealed a bi-factor configuration which, in line with what was conceptually reported by Vera et al. [16], we referred to as (the perception of) general or cognitive fatigue and (the perception of) physical fatigue. In the present study, a significant positive correlation was observed between the CIS-16R and another validated instrument for measuring WF in the country, the 3D-WFI, which adds evidence to establish the validity of the 16-item CIS in Spanish.

The nomological networks of the relationship between WF and the other variables studied in this research were in the expected direction, supporting the instrument’s validity. For example, previous studies have found relationships between task challenges (e.g., emotional and concentration demands) and work fatigue [1, 3]. This work has also found that demands on the worker in terms of emotional control, requirements for prolonged concentration, and the use of special clothing or suits for work significantly impacted WF. In this vein, using special clothing or suits for security work is a protective factor against fatigue. A security guard not wearing protective clothing breaches a basic safety rule. In addition, using special suits to protect against gunshots or other threats to physical integrity, as well as the carrying of defensive weapons, can lead to greater tranquility or stress reduction and, thus affect work fatigue. In turn, WF had a positive effect on absenteeism and a negative and significant impact on job satisfaction and health self-assessment; in other words, while job satisfaction is inversely associated with general fatigue, participants who reported more days of poor physical health also reported higher levels of physical fatigue on the CIS-16R measure. These results also align with previous research that found solid relationships between WF and absenteeism [17] and job satisfaction [16] in samples of Chilean workers. Moreover, this study has found that being male is associated with lower levels of overall work fatigue.

The availability of a validated instrument for detecting WF among Chilean workers represents a significant advancement for both applied research and occupational safety management. Its potential benefits include reducing costs associated with operational errors and workplace accidents caused by fatigue, by enabling more accurate diagnosis and the implementation of preventive interventions within organizational settings. Furthermore, such a tool can substantially contribute to strengthening public policies aimed at occupational risk prevention across various productive sectors in the country. On the other hand, the validated tool could be helpful in other Latin American countries with similar cultural and linguistic characteristics.

Regarding limitations, this research’s failure to assess the instrument’s temporal stability should be noted as a weakness. Moreover, since the sample was non-random, the study’s results cannot be generalized to the national population, as the sample is not representative. In addition, the sample’s composition is heterogeneous, with a clear predominance of participants from the security sector, while workers from the education sector represent a minority.

## Conclusions

Our proposed CIS renamed the CIS-16R to indicate the new 16-item Spanish version is an adequate instrument for assessing work fatigue in a sample of Chilean workers in the security and education sectors. Although the general reliability of its 20 original items was adequate, a detailed analysis showed that the item-total correlation of the four items was weak, so it was not appropriate to carry out a confirmatory factor analysis (CFA) under these conditions. It was, therefore, optimized as an instrument by eliminating the four items with a very weak item-scale correlation.

Regarding the CIS-16’s validity, it is composed of two sub-dimensions, which were specified in this paper to facilitate its future validation by a CFA, which should be carried out with a new sample. The bifactorial architecture discovered is consistent with what Vera et al. [16] proposed with a small sample of Chilean high-altitude miners. Thus, the CIS-16R can measure work fatigue reliably and validly in Chilean workers.

Finally, having a validated Spanish version of the CIS allows for progress in standardizing work fatigue measurement in Spanish-speaking contexts, representing a significant contribution to research and to the design of occupational health and safety interventions. Future studies could also explore its applicability across productive sectors and geographic regions.

## Data Availability

The datasets used and/or analyzed during the current study are available from the corresponding author on reasonable request.
